# Seroprevalence of Immunoglobulin-G Antibody Among Confirm Cases of COVID-19

**DOI:** 10.7759/cureus.17956

**Published:** 2021-09-14

**Authors:** Om Prakash, Bhavin Solanki, Jay K Sheth, Tejas Shah, Mina Kadam, Sheetal Vyas, Aparajita Shukla, Jayshree Pethani, Hemant Tiwari

**Affiliations:** 1 Bachelor of Medicine, Bachelor of Surgery (MBBS), Indian Administrative Services (IAS) Government of Gujarat, Ahmedabad, IND; 2 Health Department, Ahmedabad Municipal Corporation, Ahmedabad, IND; 3 Community Medicine, Ahmedabad Municipal Corporation (AMC) Medical Education Trust (MET) Medical College, Ahmedabad, IND; 4 Microbiology, Ahmedabad Municipal Corporation (AMC) Medical Education Trust (MET) Medical College, Ahmedabad, IND; 5 Community Medicine, Nathiba Hargovandas Lakhmichand (NHL) Municipal Medical College, Ahmedabad, IND; 6 Microbiology, Nathiba Hargovandas Lakhmichand (NHL) Municipal Medical College, Ahmedabad, IND

**Keywords:** severe acute respiratory syndrome - corona virus-2, covid19 cases, sero-surveillance, seroprevalence, immunoglobulin-g antibody, immunity

## Abstract

Background

Sero-surveillance to find the presence of IgG antibodies among COVID-19 cases helps in the better understanding of the immune response after COVID-19 infection.

Objectives

To estimate seropositivity among confirmed COVID-19 cases and to correlate the seropositivity with various factors affecting seropositivity.

Methods

Population-based sero-surveillance among COVID-19 cases was carried out during the second half of August 2020 in Ahmedabad using the COVID KAVACH, Immunoglobulin-G (IgG) Antibody Detection Enzyme-Linked Immunosorbent Assay (ELISA) kits. Seropositivity among cases was measured and compared with various other factors to understand the immunity status among COVID-19 cases.

Results

With 1073 positive for IgG antibodies from 1720 samples, the seropositivity among COVID-19 cases is 62.38% [95%CI 60.07-64.64%]. The difference in seropositivity based on gender was statistically not significant (Z=0.26, P=0.79). Children have the highest seropositivity (94.44%) and from young adults, to the elderly, the proportion of positivity among cases shows an increasing trend. Time gap analysis from the date of diagnosis shows that the proportion of cases with IgG antibodies increases gradually reaching its peak at around 10 weeks (third month) and then declines gradually.

Conclusion

Seropositivity among COVID-19 cases is 62.38%. The proportion of cases with IgG antibodies reaches its peak at around 10 weeks (third month) after diagnosis and then declines gradually. This fall indicates that the detected antibodies may not be long-lasting and may become undetectable/absent over a period of time. The reason for seronegative results in COVID-19 cases needs further in-depth scientific research.

## Introduction

Starting from early 2020, the pandemic of COVID-19 affected the entire world [1,2]. In view of a large number of asymptomatic cases, as also suggested by WHO, the indirect estimation of actual cases is crucial in assessing the true extent of the spread of Severe Acute Respiratory Syndrome-Coronavirus2 (SARS-CoV2) [3,4]. Sero-surveillance uncovers the asymptomatic, subclinical infection and helps in understanding the disease dynamics in a better way for planning an appropriate public health response [5,6]. Multiple sero-surveillance studies conducted during the pandemic have focused on antibodies against SARS-CoV2 in the general population [7,8]. Sero-surveillance studies among COVID-19 cases can give scientific insight. Comparison of seropositivity among cases can add additional values in the scientific knowledge & help in formulating valid predictions regarding immunity status in the post-covid period.

Ahmedabad, a city with approximately 7 million people was one of the earliest cities to witness the high caseload in the initial months of the pandemic in India. A population-based sero-surveillance was carried out during the second half of August 2020. COVID-19 cases, contacts of cases, and health care workers (HCWs) were also included as additional categories along with the general population. This article describes the sero-surveillance findings among cases of COVID-19. The primary objective was to estimate the seroprevalence among cases. The study also tried to check any association of the seropositivity with available factors like age, gender, duration from COVID-19 infection among others.

## Materials and methods

To monitor the pandemic and understand the proportion of the population already exposed to SARS-CoV2, the Indian Council of Medical Research (ICMR) issued directives to all the state governments for conducting repeated sero-surveillance studies. Health Department of the Ahmedabad Municipal Corporation (AMC) planned and conducted a population-based sero-survey. The methodological details of the study are as per the following:

Study design

This study was designed as a cross-sectional sero-surveillance study in Ahmedabad, Gujrat, India. The study population included confirmed cases of COVID-19 [based on the case definition of COVID-19, given by the World Health Organization (WHO)] [9]. The enrollment and sample collection for the study were carried out during the second half of August 2020.

Sample size calculation & sampling details

The population-based stratified sampling was used to calculate the required minimum sample size for the general population category for each of the Urban Primary Health Centre (UPHC). The earlier sero-surveillance study carried out by us in Ahmedabad showed that some of the Urban Primary Health Centre (UPHC) had nearly 50% seroprevalence [10]. Considering this, in a population of 7 million, we calculated the minimum required sample size with a 95% confidence level and a 1% margin of error. Along with the general population, COVID-19 cases, the study participants for the present study, were also enrolled separately and their sample size was kept as a minimum of 10% of the general population sample. This also ensured that the case selection is also based on the population proportion.

Ahmedabad city has 75 UPHCs spread across 48 wards and seven zones. Since the UPHCs are functional units for the COVID-19 case management, these 75 UPHCs have the details of all the reported cases from their area. To enroll a sufficient number of cases from different time periods since the pandemic, UPHC medical officers were advised to enroll cases registered during different months since the beginning of the pandemic. COVID-19 cases of either gender diagnosed at any point of time, who gave informed written consent, were enrolled as a ‘case’ through convenience sampling. However, as far as possible, an effort was specifically made to cover a wide variety of people of different age-group (with at least 10% of cases from < 18 years and > 60 years age) from different localities within the field area of the UPHC.

Testing kit & details of standardized testing

COVID KAVACH - an Anti-SARS-CoV2 Immunoglobulin-G (IgG) Antibody Detection capture Enzyme-Linked Immunosorbent Assay (ELISA) kit developed and manufactured by Zydus Diagnostics, India was used in the present study. The National Institute of Virology, Pune, India has validated the kits and the ICMR has approved their use. These kits have a sensitivity of 92.37% and a specificity of 97.9% as per the validation reports [11]. Its manufacturer claims to have no cross-reactivity with other serum samples from reverse transcription-polymerase chain reaction (RT-PCR) confirmed patients of various other infections. The manufacturer’s instructions were completely followed for the purpose of testing. To avoid sample rejection, Serum Separating Tubes (SST)-Gel Vacutee were used for the blood sample collection. Sample testing was carried out by laboratories with national-level accreditation & state of the art facilities.

Ethical considerations

The Institutional Ethics Committee of the Ahmedabad Municipal Corporation (AMC) Medical Education Trust (MET) Medical College, Ahmedabad approved the study protocol. Written informed consent was collected from all the consenting participants before enrolment. In the case of children < 18 years, an assent with the informed written consent of their parent/guardian was taken for the purpose of the study. Confidentiality was maintained as needed for the ethical conduct of the study.

Data analysis & data management

Microsoft Excel and Epi-Info were used for data analysis and data management. Our data analysis was focused on the seropositivity among cases and its correlation with available variables. Simple proportions were calculated and appropriate statistical tests were applied as necessary.

## Results

Serum samples from a total of 1722 cases (715 female & 1007 male) were collected. Out of these, two samples were rejected for insufficient quantity, one from each of the gender groups. The 1720 samples, on testing, gave negative results in 616 (35.81%), and indeterminate in 31 (1.80%) samples. The remaining 1073 results were positive for the IgG antibodies against SARS-CoV-2 with positivity of 62.38% [95% confidence interval (CI) 60.07-64.64%].

An analysis of 1720 cases (Table 1) shows that results included 714 females and 1006 males. 448 positive samples among females calculated a positivity rate of 62.75% [95% CI 59.14-66.21%] whereas 625 positive samples among males calculated a positivity rate of 62.13% [95%CI 59.09-65.07%] This difference between the two genders is statistically not significant (Z=0.26, P=0.79).

**Table 1:** Analysis of COVID-19 sero-survey positivity among cases CZ: Central Zone, EZ: East Zone, NWZ: North West Zone, NZ: North Zone, SWZ: South West Zone, SZ: South Zone, WZ: West Zone

	Female			Male			Total			
	Results	Positive	% Positivity	Results	Positive	% Positivity	Results	Positive	% Positivity	95% Confidence Interval
Total	714	448	62.75	1006	625	62.13	1720	1073	62.38	60.07 – 64.64
Age group										
0-9	7	7	100.00	11	10	90.91	18	17	94.44	72.71 – 99.86
10-19	45	22	48.89	65	36	55.38	110	58	52.73	42.98 – 62.32
20-29	145	80	55.17	182	86	47.25	327	166	50.76	45.37 – 56.14
30-39	137	69	50.36	224	139	62.05	361	208	57.62	52.47 – 62.61
40-49	152	101	66.45	205	134	65.37	357	235	65.83	60.76 – 70.55
50-59	113	81	71.68	160	100	62.50	273	181	66.30	60.36 – 71.89
60-69	79	60	75.95	107	78	72.90	186	138	74.19	67.28 – 80.32
70-79	28	21	75.00	46	38	82.61	74	59	79.73	68.78 – 88.19
80-89	8	7	87.50	5	4	80.00	13	11	84.62	54.55 – 98.08
90-99	0	0	0.00	1	0	0.00	1	0	0.00	00.00 – 00.00
Zone										
CZ	75	57	76.00	86	72	83.72	161	129	80.12	73.12 – 85.99
EZ	138	87	63.04	167	107	64.07	305	194	63.61	58.07 – 68.81
NWZ	61	34	55.74	84	42	50.00	145	76	52.41	43.96 – 60.76
NZ	99	83	83.84	140	116	82.86	239	199	83.26	77.91 – 87.77
SWZ	64	43	67.19	103	63	61.17	167	106	63.47	55.68 – 70.78
SZ	128	75	58.59	186	99	53.23	314	174	55.41	49.88 – 60.81
WZ	149	69	46.31	240	126	52.50	389	195	50.13	45.18 – 55.07

The age distribution of the cases typically follows age-heaping bias at five years gap (the table shows only grouped data) as the age of the enrolled cases were recorded without confirming any official record. The age of the cases ranged from four to 93 years with a mode of 30, a median of 40, and an average of 41.30+16.16 years. In the analysis of seropositive cases, females have a mean age of 43.25+16.76 years whereas males have a mean age of 43.15+16.36 years.

**Figure 1:**
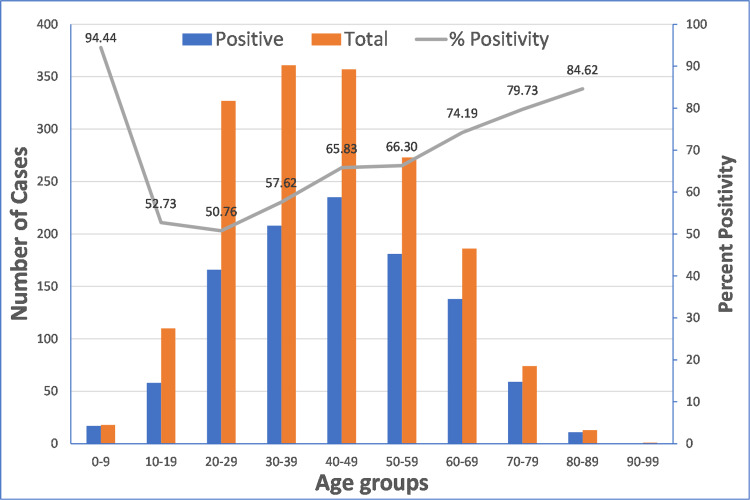
Positivity among cases based on age groups

The age of cases when grouped for 10 years period, (Figure 1) shows that 20-29 years had the lowest positivity of 50.76%. Children have the highest seropositivity (94.44%) for the 0 to nine years age group. From young adults to the elderly the seropositivity gradually increases from around 50% to around 85%. On both the extremes of age range, i.e., in children as well as the elderly, the seropositivity is high as compared to the young adults. The group data based on gender showed an almost similar trend with minor variations.

Zone-wise seropositivity ranged from 50.13% to 83.26% with the highest positivity of 83.26% in the North Zone, followed by 80.12% in the Central Zone. North Zone has more recent infections whereas Central Zone was the first and earliest zone to be affected by the pandemic.

**Figure 2:**
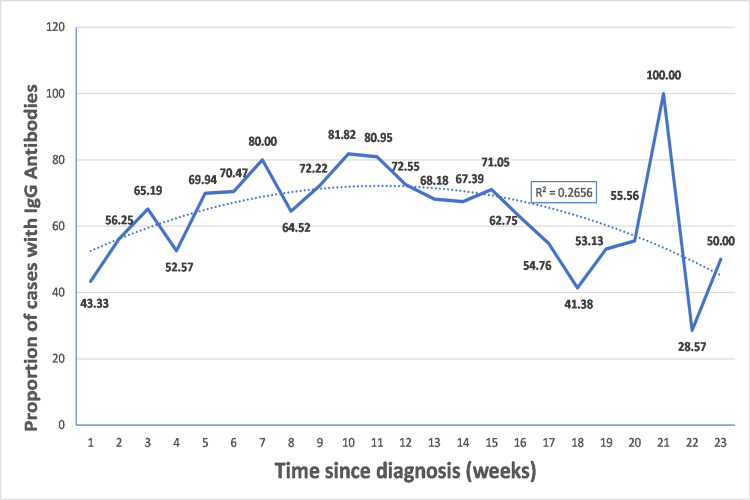
Proportion of cases with IgG antibodies based on time since diagnosis (weeks)

**Figure 3:**
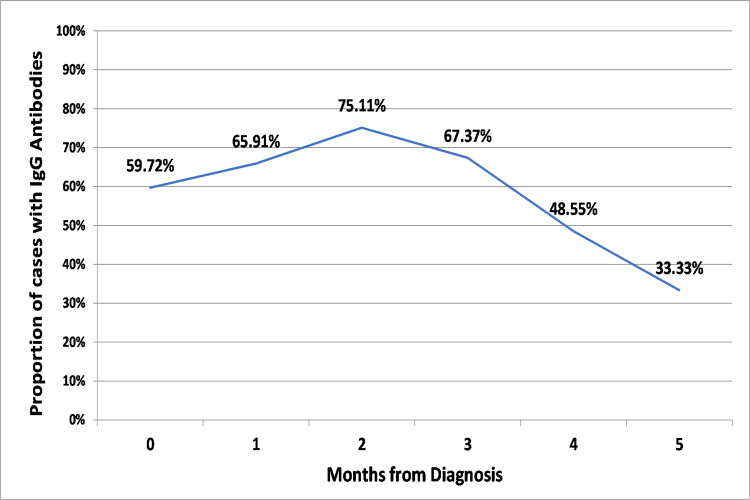
Proportion of cases with IgG antibodies based on time since diagnosis (months)

Since the seropositivity was not 100% among the cases, it was important to analyze the reasons for the same. We tried to analyze the time gap between the antibody testing and the date on which the diagnostic test for COVID-19 gave positive result. While comparing the time since diagnosis in weeks with the seropositivity (Figure 2), IgG antibodies were detected in 43.33% of cases at the first week and 56.25% at two weeks. These antibodies beyond a period of two weeks increase slowly and remain in the range of 50-80%, reaching their peak at 81.82% at around 10 weeks. The proportion of cases with IgG antibodies then gradually decreases beyond 10 weeks. The proportion of seropositive cases when compared with time since diagnosis in months (Figure 3) shows that the seropositivity among cases during the first month is 59.72%. This increases during the next two months reaching its peak of 75.11% and then falls over gradually to 33.33% over the subsequent three months.

## Discussion

Immune response after any viral infection is generally known. However, the immune response to SARS-CoV2 infection is still largely evolving [12,13]. The present study on the seropositivity among cases is probably one of the first few serological studies from India, exclusively covering the COVID-19 cases with a large sample size. Those COVID-19 cases who demonstrate the presence of IgG antibodies against SARS-CoV2 after the infection are seropositive cases. The seroprevalence in cases indicates the proportion of people who have acquired antibodies and it also indirectly indicates the proportion of cases who did not demonstrate IgG antibodies in spite of having a confirmed infection status in the past. “Should we consider this group as susceptible again for any re-infection?” is a major scientific question in the current pandemic situation. The present study also tries to highlight the proportion of cases with IgG antibodies and its correlation with the time since diagnosis, if any.

As cases are the individuals who have been infected with SARS-CoV2 in the past, one might expect all of them to have developed antibodies and expect them to be “seropositive”. However, based on our findings with the average seropositivity of 62.38% [95%CI 60.07-64.64%] among cases, it can be said that the majority of the cases demonstrate the presence of IgG antibodies after the infection with SARS-CoV2. However, it also implies that the remaining 37.62% have either not developed the antibodies, have antibodies but in undetectable proportion, or the antibodies have disappeared, after their development, during the post-covid period. Further in-depth scientific research is required for the proportion of cases that did not demonstrate the presence of IgG antibodies.

The difference in seropositivity according to gender (female cases 62.75%, male cases 62.13%) was statistically not significant (Z=0.26, P=0.79). This finding clarifies equitable risk between both the gender groups and similar findings were recorded in other studies as well [14,15]. Analysis of the age of the cases shows mean > median > mode and it indicates that the distribution of cases in our sample had many young adults as compared to the elderly and the mean is deviated on the right due to higher values of a comparatively small number of elderly cases with age more than double of the mean age.

Cases were selected randomly from UPHC/Ward through convenience sampling & it was based on population proportion and not on the reported number of cases from the respective area, So, the seropositivity among cases should not differ much. However, there is wide variation, which indicates that there may be multiple other factors affecting positivity in COVID-19 cases.

Looking at the seropositivity based on age-group-wise (Figure 1), children have the highest seropositivity. It is a scientifically documented fact that most children and young adolescents have a mild clinical illness which may be due to their strong immune response which leads to the development of IgG antibodies in this age group. On the other hand, the seropositivity of about 50% in young adults indicates that just around half of the cases from the young adult age group demonstrate IgG antibodies in the post coronavirus period. It also shows that the positivity has increasing trend as the age group increases from young adults to elderly. The documented scientific observations also show that the elderly people are more likely to have symptoms, have more severe symptoms and the symptomatic period stays for a longer duration as compared to the young adults [16-20]. This difference in clinical symptomatology may be the reason behind the higher seropositivity among the elderly as compared to young adults. The seroconversion among the asymptomatic cases is also documented to be quite low [21,22]. However, in the absence of data on clinical severity in our study, we could not check this association.

It has been documented that the onset of symptoms should be preferred rather than the date of diagnosis for consideration of this time gap [23]. However, due to the higher reliability of data, we preferred comparing the date of diagnosis rather than the onset of symptoms. Research findings to date have shown that the antibodies need some time for development after an infection, approximately one to three weeks, with an average of two weeks (14 days) [24-26]. Comparing the proportion of seropositive cases with time since diagnosis (in weeks) (Figure 2), we observed that 43.33% cases demonstrated IgG antibodies before two weeks. The seropositivity from two weeks onwards increases and reaches its peak at around 10 weeks and then gradually declines. This highlights that not every case develops IgG antibodies by two weeks and it may also take a longer time & that the detected antibodies may not stay forever; they may become undetectable/absent over a period of time. Comparison of seropositive in cases with the time since diagnosis (in months) (Figure 3), shows that the seropositivity slowly rises from 59.72% at 0 completed months to its peak of 75.11% at two completed months (61-90 days) and then slowly decline up to 33.33% over the next few months.

Both these time-gap analysis shows that IgG antibodies levels reaches its peak at 10 weeks or during the third month & then declines gradually over the next few weeks/months. This has also been documented by other studies [27-29]. This fall in the proportion of positive cases with IgG antibodies indicates that these antibodies may not be long-lasting. On the other hand, these numbers also suggest that the antibodies may still be completely absent (or undetectable) in COVID-19 cases.

## Conclusions

Seropositivity of 62.38% among COVID-19 cases suggest that all the cases may not have IgG antibodies. Among the seronegative cases, the antibodies are not developed, are undetectable, or have disappeared during the post coronavirus period. The difference in seropositivity based on gender is statistically not significant. Children have the highest seropositivity and from young adults, to the elderly, the proportion of positivity shows an increasing trend. Time gap analysis from the date of diagnosis shows that the proportion of cases with IgG antibodies increases gradually reaching its peak at around 10 weeks (third month) and then declines gradually. The reason for the seronegative results in COVID-19 cases needs further in-depth scientific research.
